# Right atrium myxoma coexisting with antiphospholipid syndrome: a case report

**DOI:** 10.1186/1476-7120-7-47

**Published:** 2009-10-11

**Authors:** Christos Pliakos, Eleni Alexiadou, Symeon Metallidis, Theodossis S Papavramidis, Stergios Kapoulas, Konstantinos Sapalidis, Pavlos Nikolaidis

**Affiliations:** 1 1st Internal Medicine Department, Aristotle University of Thessaloniki, Thessaloniki, Greece; 2 3rd Surgical Department, Aristotle University of Thessaloniki, Thessaloniki, Greece

## Abstract

In this case report we describe a rare case of right atrium myxoma that coexisted with antiphospholipid syndrome in a young woman. We describe the unusual findings and diagnostic challenges combined with a review of the literature.

## Background

Myxoma is the most common type of primary cardiac tumors and is usually localized in the left atrium. A right atrium myxoma is rarely observed. Its clinical presentation is ranging from an asymptomatic condition or constitutional symptoms to cardiac obstruction or pulmonary embolism. On the other hand, antiphospholipid syndrome is related to thrombotic conditions and the finding of a cardiac mass would be expected to be a thrombus. A comprehensive review of the literature reveals a limited number of cases with coexistence of cardiac myxoma and antiphospholipid syndrome.

## Case report

A 28-year old female patient was admitted to our hospital because of two-day nausea and pain on the right upper quadrant of the abdomen. It was mentioned, in her medical history, an episode of acute but transient vision loss that lasted for a day, five years ago.

On admission, blood pressure was 130/85 mmHg, heart rate 84 beats/min, respiratory rate 16 breaths/min and temperature was normal. There were no clinical findings on cardiac auscultation. Except of mild pain on the right upper quadrant, the rest of the physical examination was unremarkable.

Laboratory tests revealed the following results: WBC = 11,6 K/μL, Hb = 13,7 g/dL, Ht = 39,9%, PTL = 95 K/μL, LDH = 384 U/l, AST = 16 U/l, ALT = 16 U/l, γGT = 10 U/l, ALP = 58 U/l, bilirubin = 0,73 mg/dl, CRP = 0,07 mg/dl, ESR = 40 mm and urine test within normal limits. Chest X-ray didn't show any pathologic finding.

On further investigation, ultrasonography and computed tomography of the abdominal cavity were obtained and identified the presence of free fluid around the liver.

On the second day of the nursery, she had low-grade fever but her clinical condition worsened, with abdominal tenderness, hypotension and tachycardia. The patient was transferred to the surgical clinic and underwent investigational laparotomy. There were no special findings, appendectomy was performed and the histological examination of the appendix, a mesenteric lymph node revealed only signs of inflammation.

**Figure 1:**
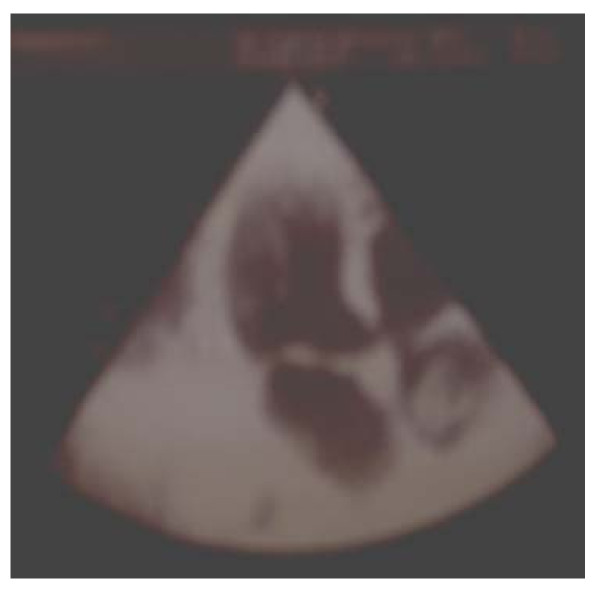
**Echocardiography of a right atrial mass, 2 × 2 cm, attached to the atrial septum**.

Two days after the surgery, the patient developed tachypnea, tachycardia and fever (temperature of 38,8°C). A computed tomographic scan of the chest and the abdomen was obtained as well as a transthorasic echocardiogram in order to rule out pulmonary embolism. The first imaging study showed infiltrations of the right lower lobe accompanied by pleural effusion, while the echocardiogram revealed the presence of a cardiac mass in the right atrium, mobile and attached to the interatrial septum (figure 1). Differential diagnosis was between thrombi and myxoma. Low-molecular-weight heparin was administrated to the patient as a therapy for pulmonary embolism.

During her nursery, it was observed that she had steadily worsening thrombocytopenia and prolonged aPTT, so more thorough blood tests were done as far as coagulant disorders are concerned. Based on lupus anticoagulant (LA) positivity, anticardiolipin (aCL) antibodies and β_2_GPI remarkable elevated, the diagnosis of antiphospholipid syndrome was suggested. Because patients with antiphospholipid syndrome often develop thrombi it was thought that the mass in the right atrium was a thrombi.

**Figure 2:**
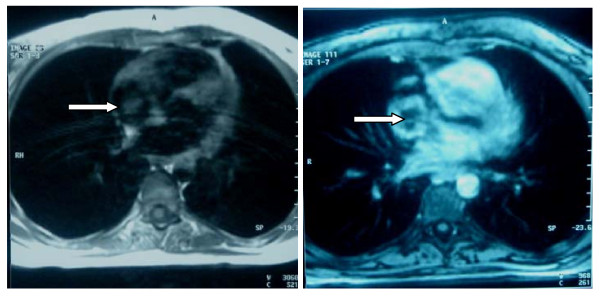
**MRI showing a mass occupying the right atrium**.

The patient underwent a magnetic resonance imaging, as a more helpful imaging technique for surgical planning in order to remove the thrombi. Not only the size and the location of the cardiac mass were better depicted, but also pulmonary emboli were revealed on both lower lobes, especially on the right one (figure 2).

The mass was successfully excised and the histological examination confirmed the macroscopic diagnosis of myxoma and not a thrombi. She had a good postoperative course and was discharged with oral anticoagulant therapy maintaining an international normalized ratio (INR) between 2,5 and 3. Follow-up echocardiograms showed no recurrence of the myxoma and repeated blood tests after 12 weeks confirmed the diagnosis of antiphospholipid syndrome.

## Conclusion

Cardiac tumors, either primary or secondary, are uncommon, reported in 0,056% to 1,23% in autopsy series [1]. Primary cardiac tumors are accounting for 76%, in a multicentre retrospective analysis [2], with 75% of these to be benign and 50% of the last group to be myxomas. They occur mainly in women, between the third and sixth decades of life [3] and they may develop in any chamber of the heart. The majority is situated in the atria (75% in the left atrium), with only 5-10% presented in the ventricles [4]. Atypical locations and multiple myxomas occur in familiar cases [5].

Macroscopically, there are two types of myxomas: a solid and round shaped with a nonmobile surface and a polypoid one with an asymmetrical, soft and mobile surface [6]. It is thought that multipotential mesenchymal cells from embryonic rests give rise to myxomas [4]. Histologically, they consist of a mixoid matrix composed of an acid-mucopolysaccharide-rich stroma and polygonal cells with scant eosinophillic cytoplasm throughout it. They may, also, contain cysts, calcifications, areas of haemorrhage or extra-medullary heamatopoiesis as well as gland-like elements [7].

The clinical features depend on the size, the location, the mobility and the fragility of the mass and they may be constitutional, obstructive or embolic. The constitutional symptoms, such as fever, weight loss, anemia, arthralgias and rash, rely on an immunologic response with an increase of CRP, IL-6 and γ-globulin [8] due to bleeding and degeneration within the mass and releasing tumor fragments. The laboratory tests may mimic those of autoimmune and rheumatic diseases [9] and this is usually the main reason for delaying the diagnosis of a myxoma [6]. In case of a right atrial myxoma, the obstruction of tricuspid valve can give rise to signs of right-sided heart failure, such as dyspnea, cyanosis, general edema or syncope [10]. Moreover, the blockage of the vena cava can result in a Budd-Chiari syndrome with acute abdominal pain [11]. The most important life-threatening complication is pulmonary embolism and it is demonstrated in 40% of patients with cardiac tumors. Rarely, it has been reported cases of infected right atrial myxomas [12].

As far as the diagnosis is concerned, transthoracic echocardiophy is the method of choice for the detection and description of the size, shape, location, attachment to the cardiac wall and movement of the myxoma. Transesophageal echocardiography should be considered in case of clinical suspicion and nondiagnostic transthoracic echocardiography. Magnetic resonance imaging (MRI) is a quite expensive but more accurate technique in determining the structure and special tissue characteristics of the myxoma, αποτελώντας an important role in surgical planning [13].

The differential diagnosis of a right atrial lession includes both benign (myxoma, thrombus and vegetations) and malignant masses and the confirmation of the diagnosis is the histopathologic examination.

Therapeutic approach consists only of surgical excision of the mass as soon as possible, especially on its symptomatic forms of embolism and heart failure. A better preoperative assessment can allow a minimally invasive approach with video-thoracoscopy [14]. The intra-operative mortality rate is low and the short and long-term prognosis excellent. Follow-up echocardiography is needed to rule out cases of recurrence.

The second clinical condition that was present in our case report was the antiphospholipid syndrome (APS) which diagnosis was established six weeks after the surgical removal of the myxoma. This syndrome is a disorder characterized by recurrent venous or arterial thrombosis and/or fetal losses associated with characteristic laboratory abnormalities, such as persistently elevated levels of antibodies directed against membrane anionic phospholipids (anticardiolipin antibody, antiphosphatidylserine) or their associated plasma proteins, predominantly beta-2 glycoprotein I (β_2_GPI), or evidence of a circulating anticoagulant. It is presented either as primary or secondary to rheumatic or autoimmune disorders, mainly systemic lupus erythematosus (SLE) and has been documented a young female predominance.

The diagnostic criteria are clinical and laboratory ones. The clinical criteria are: 1) one or more confirmed vascular thrombotic episode, and 2) pregnancy morbidity. The laboratory criteria are: elevated levels of 1) aCL antibodies, 2) β_2_GPI, 3) LA, on at least 2 occasions at least 12 weeks apart. There should be at least one clinical and one laboratory criterion present for the diagnosis [15].

The medical management of the syndrome is anticoagulant therapy with oral vitamin K antagonists and INR between 2 and 3, so as to eliminate the thrombosis risk. In cases of high levels of antibodies but no symptoms, patients should be treated with low-dose aspirin [16].

Usually patients have only one disease that can explain their symptoms. In our case report the patient had two different clinical conditions that were responsible for her symptoms. We can only suggest what was responsible for her onset clinical symptoms that led to diagnostic laparotomy. Some authors have reported that the blockage of the vena cava by a myxoma, can result in a Budd-Chiari syndrome with acute abdominal pain. This scenario can provide a reasonable explanation but we were not able to prove it. The other question that rises reasonably is that if there was a connection between the myxoma and the presence of antiphospholipid syndrome. While reviewing the literature, only Quintanilla et al. has reported a similar case and suggested that that interleukin-6 produced by the myxoma could trigger an immunological reaction leading to the primary antiphospholipid syndrome [17]. We have to emphasize that there was no evidence of antiphospholipid syndrome relapse after the myxoma excision. On the other hand many cases of APS in the literature have reported cardiac thrombi mimicking myxoma [18]. In our case report if the myxoma was smaller it would result in a delay of diagnosis, because no surgery would be preformed for the myxoma excision.

In conclusion, it should be mentioned that even though both right atrial myxoma and antiphospholipid syndrome are uncommon conditions, separately or not, there should be always clinical suspicion in order to prevent their serious and probably fatal complications.

## Consent

Written informed consent was obtained from the patient for publication of this case report and any accompanying images. A copy of the written consent is available for review by the Editor-in-Chief of this journal.

## Competing interests

The authors declare that they have no competing interests.

## Authors' contributions

PC was the cardiologist that diagnosed the myxoma and participated in the drafting of the manuscript. SK, KS and PT have been involved in drafting the manuscript. MS and AE reviewed the literature and helped to draft the manuscript. NP has given final approval of the version to be published. All authors read and approved the final manuscript.
